# Novaes EMDF, Aquino EML, Gabrielli L, Matos SMA, Griep RH, Fonseca MJM, et al. Percepção de imagem corporal, características socioeconômicas e estilo de vida em mulheres participantes do ELSA-Brasil na Bahia, Brasil. Cad Saúde Pública 2024; 40(2):e00107823.

**DOI:** 10.1590/0102-311XER107823

**Published:** 2024-04-22

Onde se lê:

**Figura 1:**
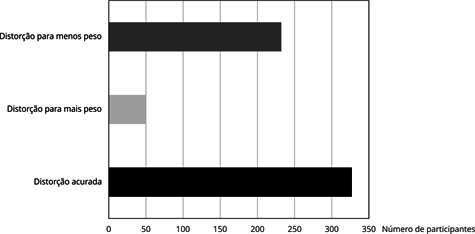
Distribuição das participantes do ELSA-Brasil (*Estudo Longitudinal da Saúde do Adulto*), residentes na Bahia, Brasil, segundo percepção da imagem corporal (2012-2014).

Leia-se:

**Figura 1:**
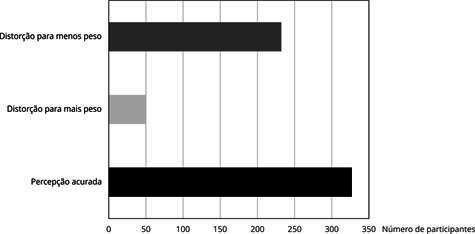
Distribuição das participantes do ELSA-Brasil (*Estudo Longitudinal da Saúde do Adulto*), residentes na Bahia, Brasil, segundo percepção da imagem corporal (2012-2014).

